# The Emerging Field of Epitranscriptomics in Neurodevelopmental and Neuronal Disorders

**DOI:** 10.3389/fbioe.2018.00046

**Published:** 2018-04-13

**Authors:** Margarita T. Angelova, Dilyana G. Dimitrova, Nadja Dinges, Tina Lence, Lina Worpenberg, Clément Carré, Jean-Yves Roignant

**Affiliations:** ^1^Drosophila Genetics and Epigenetics, Sorbonne Université, Centre National de la Recherche Scientifique, Biologie du Développement—Institut de Biologie Paris Seine, Paris, France; ^2^Laboratory of RNA Epigenetics, Institute of Molecular Biology, Mainz, Germany

**Keywords:** RNA modification, m^5^C, Nm, pseudouridine, m^6^A, neurons, disease

## Abstract

Analogous to DNA methylation and histone modifications, RNA modifications represent a novel layer of regulation of gene expression. The dynamic nature and increasing number of RNA modifications offer new possibilities to rapidly alter gene expression upon specific environmental changes. Recent lines of evidence indicate that modified RNA molecules and associated complexes regulating and “reading” RNA modifications play key roles in the nervous system of several organisms, controlling both, its development and function. Mutations in several human genes that modify transfer RNA (tRNA) have been linked to neurological disorders, in particular to intellectual disability. Loss of RNA modifications alters the stability of tRNA, resulting in reduced translation efficiency and generation of tRNA fragments, which can interfere with neuronal functions. Modifications present on messenger RNAs (mRNAs) also play important roles during brain development. They contribute to neuronal growth and regeneration as well as to the local regulation of synaptic functions. Hence, potential combinatorial effects of RNA modifications on different classes of RNA may represent a novel code to dynamically fine tune gene expression during brain function. Here we discuss the recent findings demonstrating the impact of modified RNAs on neuronal processes and disorders.

## Introduction

An estimated 1–2% of all genes in a given organism contribute to nucleic acid modification systems, suggesting biological importance of modified nucleotides (Grosjean, 2009). A classic example is the methylation of cytosine on DNA, which acts as a critical epigenetic regulator of gene expression (Bird, 2002). Additionally, current advances in RNA modification research report over 140 distinct post-transcriptional RNA modifications (Cantara et al., 2011; Machnicka et al., 2013). Initial knowledge has been derived from studies on abundant non-coding RNAs (ncRNAs), such as transfer RNAs (tRNAs) and ribosomal RNAs (rRNAs), in prokaryotes and simple eukaryotes. These pioneer investigations described a diverse, chemically complex, and strongly conserved nature of RNA nucleotide modifications (Cantara et al., 2011; Machnicka et al., 2013). The most heavily modified RNAs in any cell type and organism are tRNAs. Up to 20% of nucleotides in mammalian cytoplasmic tRNAs carry modifications (Motorin and Helm, 2011; Pan, 2018). Modified nucleotides outside the anticodon loop of tRNAs occur non-randomly at conserved positions across diverse species and affect in general its stability (Helm, 2006; Motorin and Helm, 2010). In addition, modifications in the anticodon loop can contribute to optimize mRNA decoding by directly affecting codon-anticodon interactions (Agris, 2008).

Aberrant tRNA and rRNA modifications have been linked to various human disease syndromes and the phenotypes are often observed in specific tissues such as the gonads and the nervous system (Torres et al., 2014). Notably, increasing number of predicted human transfer RNA (tRNA) modification genes have been associated with neurological disorders, in particular with intellectual disability (ID) (for recent review see Bednárová et al., 2017). ID, or previously known as Mental Retardation (MR), is characterized by non-progressive cognitive impairment and affects 1–3% of the general population (Daily et al., 2000). It is presently unclear whether all observed phenotypes are caused by aberrant tRNA modifications, by effects on unidentified other RNA substrates (see below) and/or by a modification-independent function of the involved enzymes (Guo and Schimmel, 2013; Genenncher et al., 2018). Likewise, it is unknown why some tissues, in particular the brain, are more sensitive to the loss of these modifications.

Importantly, besides the heavily modified tRNAs and rRNAs, mRNAs, small and long non-coding RNAs were also found to harbor post-transcriptional modifications. Recent technological advances that allowed mapping of selected RNA modifications on a transcriptome-wide scale revealed widespread distribution of N6-methyladenosine (m^6^A), pseudouridine (Ψ) and ribose 2′-O-methylation (Nm) on mRNA (Dominissini et al., 2012; Meyer et al., 2012; Carlile et al., 2014; Schwartz et al., 2014a; Dai et al., 2017). The prevalence of some others, including N1-methyladenine (m^1^A) and 5-methylcytidine (m^5^C) is still debated (Dominissini et al., 2016; Li et al., 2016, 2017c; Dominissini and Rechavi, 2017; Legrand et al., 2017; Safra et al., 2017). m^6^A, the most abundant mRNA modification, was shown to affect almost every step of mRNA biogenesis, including splicing, export, translation, and mRNA decay (Lence et al., 2017; Roignant and Soller, 2017). It is thus not surprising that misregulation of m^6^A results in several physiological defects, including brain development abnormalities, obesity, cancer, and other diseases (Batista, 2017; Dai et al., 2018). In addition, the discovery of m^6^A RNA demethylases (Jia et al., 2011; Zheng et al., 2013; Jacob-Hirsch et al., 2018) and the identification of m^6^A-binding proteins (Dominissini et al., 2012) indicated that similarly to DNA modification, RNA methylation can be reversible and convey information *via* recognition of effector proteins.

Altogether these recent studies revealed an entire new layer of regulation of gene expression, which has been central to the development of a novel concept called “RNA epigenetics or epitranscriptomics” (He, 2010; Meyer et al., 2012). However, the exact biological function of the majority of modified RNA nucleotides remains to be discovered. In this review, we will focus on several RNA modifications and will discuss their involvement in the development of the brain and neurological disorders.

## 5-methylcytosine (m^5^C)

**Figure 1:**
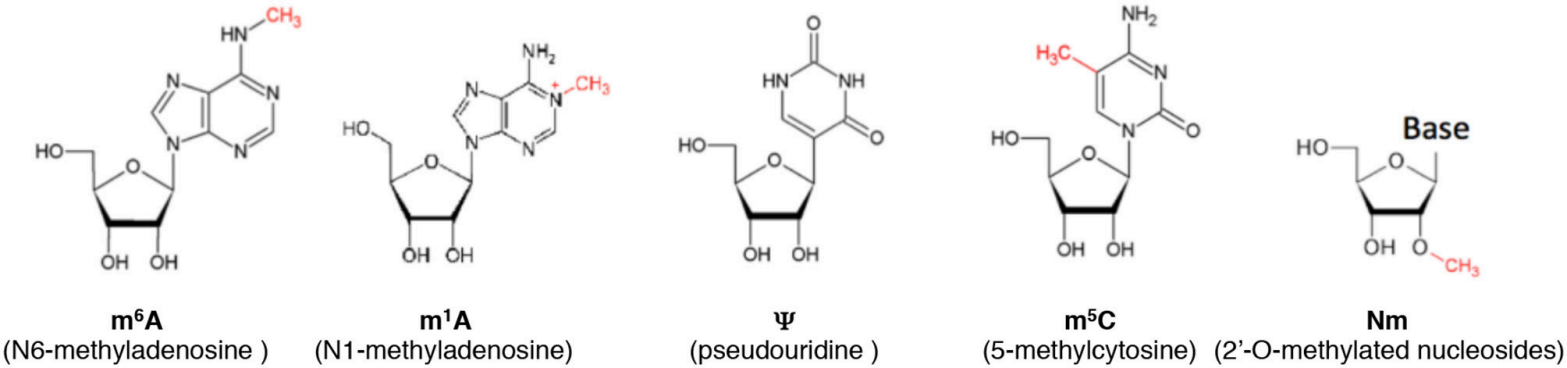
Some of the most common modifications in RNA.

Cytosine can be methylated at the 5^th^ position of the pyrimidine ring to form 5-methylcytosine (m^5^C) (Figure 1). Various eukaryotic cytosine-5-RNA methyltransferases catalyze the formation of m^5^C at specific positions (Motorin and Grosjean, 1999; Brzezicha et al., 2006; Sharma et al., 2013; Metodiev et al., 2014; Haag et al., 2015; Schosserer et al., 2015). The analysis of genetic mutations in two particular RNA cytosine-5 methyltransferase family members (Dnmt2/Trdmt and NCL1/TRM4/NSun2) has provided important insights into the biological effects of aberrant m^5^C deposition.

### Dnmt2

Dnmt2 is a member of the most widely conserved eukaryotic cytosine-5-DNA methyltransferase protein family (Goll and Bestor, 2005). Despite this classification, only few studies reported Dnmt2-mediated DNA methylation (Hermann et al., 2003; Kunert et al., 2003; Phalke et al., 2009) and it is today acknowledged that Dnmt2 functions mainly as a tRNA methylase (Okano et al., 1998; Schaefer and Lyko, 2010a,b; Raddatz et al., 2013). Dnmt2 methylation activity on position C38 of three tRNAs, which include tRNA^Asp^, tRNA^Val^, and tRNA^Gly^, has been described in yeast, *Drosophila*, mouse, and human cells (Goll et al., 2006; Jurkowski et al., 2008; Schaefer et al., 2010). Knockdown of Dnmt2 in zebrafish embryos leads to differentiation defects in some organs, and notably, to abnormal neurogenesis in the hypothalamus and diencephalon (Rai et al., 2007). In *Dnmt2* mutant flies, reduced viability under stress conditions was observed (Schaefer et al., 2010). This is in accordance with previous studies that suggest an increased tolerance for stress in *Drosophila* and *Entamoeba* upon Dnmt2 overexpression (Lin et al., 2005; Fisher et al., 2006). Nevertheless, the majority of studies suggests that *Dnmt2* mutation does not trigger strong detrimental phenotypes in yeast, *Drosophila* and mice (Wilkinson et al., 1995; Kunert et al., 2003; Goll et al., 2006; Schaefer et al., 2010), which raises the question why zebrafish relies on Dnmt2 for proper development, whereas mice and flies do not. One possible explanation is that these organisms have redundant mechanisms that compensate for the loss of Dnmt2, which may be absent or less robust in zebrafish. Consistent with this possibility, it was shown that *Dnmt2* mutant mice exhibit lethal phenotypes in the absence of a second m^5^C methyltransferase, NSun2 (Tuorto et al., 2012). In human, polymorphisms in *DNMT2* have been associated with spina bifida, a congenital malformation of the central nervous system (Franke et al., 2009).

### NSun2

Unlike Dnmt2, it has been established that mammalian NSun2 does not only modify tRNAs (Blanco et al., 2011; Tuorto et al., 2012) but also other small ncRNAs such as 7SK, vault, and Y-RNAs (Hussain et al., 2013; Khoddami and Cairns, 2013). *Dnmt2* and *Nsun2* double knockout mice showed a lethal phenotype. However, deletion of *NSun2* alone (Blanco et al., 2011; Tuorto et al., 2012; Hussain et al., 2013) or in combination with *Dnmt2* (Rai et al., 2007; Tuorto et al., 2012) in specific tissues impairs cellular differentiation pathways in mammalian skin, testes, and brain. The function in the brain appears conserved as *Nsun2* mutations are associated with ID and Dubowitz-like syndrome in humans (Abbasi-Moheb et al., 2012; Khan et al., 2012; Martinez et al., 2012), as well as with microcephaly in human and mice (Blanco et al., 2014). In addition, a recent study in human and mice neuron precursor cells showed that m^5^C deposited by Nsun2 regulates neural stem cell (NSC) differentiation and motility (Flores et al., 2017). This study thus provides some links between the failure of RNA m^5^C deposition and the associated brain development diseases.

It is intriguing that patient fibroblasts and *Nsun2*-deficient mice (Blanco et al., 2014), as well as *Dnmt2* mutant flies (Durdevic et al., 2013), exhibit increase cleavage of tRNA, and elevated production of tRNA fragments (tRFs). This accumulation of tRFs reduces protein translation rates and increases oxidative stress as well as neuronal apoptosis. Interestingly, reducing tRNA cleavage in *Nsun2*-deficient brains is sufficient to rescue sensitivity to oxidative stress, implying that tRFs play a role in Nsun2-mediated defects.

## (2′-O)-methylation (Nm)

2′-O-methylation (Nm) is a common nucleoside modification of RNA, where a methyl group is added to the 2′ hydroxyl of the ribose moiety (Figure 1). Nm increases hydrophobicity, protects RNAs from nuclease attacks and stabilizes helical structures (Kurth and Mochizuki, 2009; Byszewska et al., 2014; Kumar et al., 2014; Yildirim et al., 2014). Nm is predominantly found internally in ribosomal RNAs and small nuclear RNAs as well as in tRNAs and in a number of sites on mRNA (Darzacq et al., 2002; Rebane et al., 2002; Kurth and Mochizuki, 2009; Zhao et al., 2012; Somme et al., 2014; Dai et al., 2017). This modification is also present at the 3′-end of miRNAs and siRNAs in plants (Li et al., 2005; Yu et al., 2005), as well as in siRNAs and piRNAs in animals (Horwich et al., 2007; Saito et al., 2007). Nm methyltransferases acting on tRNAs are highly conserved from bacteria and archaea to humans (Somme et al., 2014) and usually target positions in the anticodon loop. For instance, TRM7 in *S. cerevisiae* modifies positions 32 and 34 of selected tRNA, amongst which is tRNA^Phe^ (Pintard et al., 2002; Guy et al., 2012). Strikingly, FTSJ1, the TRM7 ortholog in human, methylates the exact same positions of the exact same tRNAs (Guy and Phizicky, 2015; Guy et al., 2015). Consistent with a conserved function, expression of human FTSJ1 can suppress the severe growth defect of *S. cerevisiae* Δ*trm7* mutants (Pintard et al., 2002; Guy and Phizicky, 2015). Reduction of the modification level in tRNA^Phe^ was reported in carcinoma and neuroblastoma in mice (Pergolizzi and Grunberger, 1980; Kuchino et al., 1982) and is associated with ID in human (see below).

### FTSJ1

One of the best characterized associations between ID in human and mutations in a gene encoding for Nm is the one between non-syndromic X-linked ID (NSXLID) and mutations in the *FTSJ1* gene (OMIM:300499) (Guy et al., 2015). One third of the X-linked ID (XLID) conditions are syndromic (S-XLID) and the other two thirds are non-syndromic (NS-XLID) (Lubs et al., 2012). NSXLID is associated with no obvious and consistent phenotype other than mental retardation (IQ < 70), indeed NSXLID disorders are clinically diverse and genetically heterogeneous. *FTSJ1* loss of function causes NSXLID retardation in males (Froyen et al., 2007; Takano et al., 2008). Heterozygous loss of function mutations in females do not cause the disease, which is probably due to inactivation of the affected X chromosome. Several alleles of *FTSJ1* from six independent families correlate with NSXLID. All of these alleles lead to a reduction in mRNA levels and/or protein function (Willems et al., 1993; Hamel et al., 1999; Freude et al., 2004; Ramser et al., 2004; Froyen et al., 2007; Takano et al., 2008; Guy et al., 2015; Table 1). Consistently with the 2′-O-methyltransferase activity of FTSJ1 on tRNAs, Guy and Phizicky reported that two genetically independent lymphoblastoid cell lines (LCLs) of NSXLID patients with *FTSJ1* loss of function mutations nearly completely lack Cm_32_ and Gm_34_ on tRNA^Phe^ (Guy et al., 2015). Additionally, tRNA^Phe^ from a patient carrying an *FTSJ1-p.A26P* missense allele specifically lacks Gm_34_, but has normal levels of Cm_32_. tRNA^Phe^ from the corresponding *Saccharomyces cerevisiae TRM7-A26P* mutant also specifically lacks Gm_34_. Altogether, these findings strongly suggest that the absence of Gm_34_, but not Cm_32_ modification on tRNA^Phe^ causes NSXLID in patients carrying distinct *FTSJ1* alleles. Nevertheless, the molecular consequences arising from the loss of this 2′-O-methylation are not yet determined. Furthermore, it is noteworthy to mention two additional studies involving families from the Chinese Han population (Dai et al., 2008; Gong et al., 2008), where three single nucleotide polymorphisms (SNPs) in the *FTSJ1* gene were analyzed. Authors found a positive association with occurrence of NSXLID (Dai et al., 2008) as well as with general cognitive ability, verbal comprehension, and perceptual organization in male individuals (Gong et al., 2008). Although it seems tempting to link the variance of *FTSJ1* gene to general human cognitive ability, more profound studies are needed to support this idea.

**Table 1:** Proteins required for writing, reading, or removal of different RNA modifications and their mutations associated with altered brain functions.

**Modification**	**Gene**	**Organism**	**Defect**	**Effect/Disease**	**References**
m^5^C	Dnmt2/Trdmt	*Dr*	KD	Abnormal neurogenesis in the hypothalamus and diencephalon. Defects in retina and liver.	Rai et al., 2007
		*Dm*	Lof	Decreased tolerance to stress (reduced viability under stress conditions).	Schaefer et al., 2010
	NSun2/NCL1/TRM4	*Hs*	Lof	Autosomal-recessive ID, facial dysmorphism, microcephaly and Dubowitz-like syndrome.	Abbasi-Moheb et al., 2012; Khan et al., 2012; Martinez et al., 2012
		*Mm*	Lof	Impaired cortical, hippocampal and striatal expansion during development Microcephaly. Decrease in neural stem cell (NSC) differentiation and motility.	Blanco et al., 2014; Flores et al., 2017
		*Dm*	Lof	Severe short-term memory deficits.	Abbasi-Moheb et al., 2012
	Dnmt2, NSUN2 double mutant	*Ms*	KO	Reduced proliferation rates, underdeveloped pheno- type in several tissues, including thickness and organization of the cerebral cortex.	Tuorto et al., 2012
Nm	FTSJ1	*Hs*	Lof	Nonsyndromic X-linked ID (NSXLID) in males.	Willems et al., 1993; Hamel et al., 1999; Freude et al., 2004; Guy et al., 2015
			SNPs	Impact on general cognitive ability, verbal comprehension, and perceptual organization in males.	Gong et al., 2008
			Gof	ID	Giorda et al., 2009; Honda et al., 2010
	TRMT44	*Hs*	SNPs	Partial epilepsy with pericentral spikes (PEPS).	Leschziner et al., 2011
	C/D box snoRNAs SNORD115 (HBII-52); SNORD116 (HBII-85) and others in the 15q11-q13 region	*Hs*	Lof	Prader-Willi syndrome (PWS).	Cavaillé et al., 2000; Kishore and Stamm, 2006; Peters, 2008; Sahoo et al., 2008; Sridhar et al., 2008; Doe et al., 2009; Duker et al., 2010
	15q11-q13 region	*Hs*	Gof	Autism	Bolton et al., 2004; Cook and Scherer, 2008
	Hen1	*Dm*	Lof	Accelerated neurodegeneration-related phenotypes (brain vacuolization, memory defaults and shorter life span).	Abe et al., 2014
Ψ	Unknown	*Hs*	Gof	Alzheimer's disease	Lee et al., 2007
		*Hs*	Lof	Myotonic dystrophy type 2 (DM2).	Delorimier et al., 2017
	Pus1 (TruA family member)	*Hs*	Lof	Mild-cognitive impairment, mitochondrial myopathy and sideroblastic anemia.	Cao et al., 2016
	Pus3 (TruA family member)	*Hs*	Lof	ID	Shaheen et al., 2016
	DKC1 (dyskerin)	*Hs*	Lof	X-linked recessive dyskeratosis congenita (DKC)	Heiss et al., 1998
m^6^A	METTL3 (m^6^A writer)	*Hs*	Lof	Impaired neuronal differentiation and formation of mature neurons from embryoid bodies.	Batista et al., 2014; Geula et al., 2015
		*Dm*	SNPs	Severe locomotion defects due to altered neuronal functions.	Haussmann et al., 2016; Lence et al., 2016; Kan et al., 2017
	Mettl14 (m^6^A writer)	*Mm*	cKO	Delayed specification of different neuronal subtypes during brain development. Altered axon regeneration.	Yoon et al., 2017; Wang et al., 2018; Weng et al., 2018
	Wtap (m^6^A writer)	*Dr*	KD	Smaller brain ventricles and curved notochord.	Ping et al., 2014
	ZC3H13	*Hs*	SNP	Schizophrenia	Oldmeadow et al., 2014
	Spenito (m^6^A writer)	*Dm*	Lof	Control axon outgrowth, branching and synaptic bouton formation.	Gu et al., 2017
	Ythdc1 (m^6^A reader)	*Dm*	KD	Enhancement of SCA1-induced neurodegeneration	Fernandez-Funez et al., 2000
	ALKBH5 (m^6^A eraser)	*Hs*	SNP	Major depressive disorder (MDD).	Du et al., 2015
	FTO (m^6^A eraser)	*Hs*	SNP	Decreased brain volume, increased risk for attention-deficit/hyperactivity disorder (ADHD) and Alzheimer's disease.	Keller et al., 2011; Reitz et al., 2012; Choudhry et al., 2013; Melka et al., 2013; Li et al., 2017a
		*Mm*	KO	Altered behavior (e.g., locomotion defects), abnormal electrophysiological response to cocaine (impaired dopamine type 2 and 3 receptor response) and enhanced consolidation of cued fear memory.	Hess et al., 2013; Widagdo et al., 2016

*ID, intellectual disability; KD, knockdown; KO, knock out; cKO, conditional knock out; Lof, reduced function or loss of function; Gof, gain of function; SNP, single nucleotide polymorphism; Hs, Homo sapiens; Mm, Mus musculus; Dr, Danio rerio; Dm, Drosophila melanogaster N6-methyladenosine (m^6^A); pseudouridine (Ψ), 5-methylcytosine (m^5^C); and 2′-O-methylation (Nm)*.

### TRMT44

TRMT44 is a putative 2′-O-methyluridine methyltransferase predicted to methylate residue 44 in tRNA^Ser^ (Leschziner et al., 2011). Mutations in this gene were identified as a causative mutation in partial epilepsy with pericentral spikes (PEPS), a novel mendelian idiopathic epilepsy (Leschziner et al., 2011). However, the underlying mechanisms are currently unknown.

### Small nucleolar RNAs (snoRNAs)

snoRNAs are a class of regulatory RNAs responsible for post-transcriptional modification of ribosomal RNAs (rRNAs). Two families of snoRNAs have been described, based on their structure and function: C/D box snoRNAs are responsible for 2′-O-methylation (Cavaillé et al., 1996), whereas H/ACA box snoRNAs mediate pseudouridylation (Ganot et al., 1997 and see the following chapter in this review). In zebrafish, loss of three snoRNAs results in impaired rRNA modifications, causing severe developmental defects including growth delay and deformations in the head region (Higa-Nakamine et al., 2012). In human, C/D box snoRNAs have been implicated in Prader-Willi syndrome (PWS), a complex neurological disease characterized with mental retardation, low height, obesity, and muscle hypotonia (Sridhar et al., 2008; Doe et al., 2009). In several independent studies, PWS was shown to be caused by the loss of imprinted snoRNAs in locus 15q11-q13. Large deletions of this region underlie about 70% of cases of PWS (Peters, 2008), whereas duplication of the same region is associated with autism (Belmonte et al., 2004; Bolton et al., 2004; Cook and Scherer, 2008). Locus 15q11–q13 contains numerous copies of two C/D box snoRNAs—SNORD115 (HBII-52), and SNORD116 (HBII-85) (Cavaillé et al., 2000). SNORD115 is believed to play key roles in the fine-tuning of serotonin receptor (5-HT2C) by influencing its pre-mRNA splicing (Vitali et al., 2005; Kishore and Stamm, 2006; Falaleeva et al., 2017), whereas SNORD116 loss is thought to contribute to the etiology of the PWS (Cavaillé et al., 2000; Sahoo et al., 2008; Duker et al., 2010).

### Hen1/Pimet

Hen1/Pimet is a conserved enzyme, which adds 2′-O-methyl group to 3′-terminal nucleotides of miRNAs and siRNAs in plants, and of siRNAs and piRNAs in animals. Addition of this modification protects these small non-coding RNAs (sncRNAs) from 3′ → 5′ exonuclease degradation (Li et al., 2005; Horwich et al., 2007; Saito et al., 2007; Terrazas and Kool, 2009; Ross et al., 2014). In the absence of Hen1/Pimet, piRNA, and siRNA are destabilized and sncRNA silencing activities are compromised. Surprisingly, *Hen1* mutant flies display neither increased lethality nor sterility under normal laboratory conditions but show however accelerated neurodegeneration (brain vacuolization), memory default, and shorter lifespan (Abe et al., 2014). This suggests a protective effect of Nm and small RNA pathways against age-associated neurodegenerative events. Accordingly, *Drosophila* lacking the siRNA effector, Argonaute 2 (Ago2), are viable but exhibit memory impairment and shortened lifespan (Li et al., 2013).

## Pseudouridine (ψ)

Pseudouridine (also known as 5-ribosyluracil or ψ) is the first discovered (Cohn and Volkin, 1951) and most abundant RNA modification, present in a broad range of non-coding RNA, and was also recently detected in coding mRNA (Carlile et al., 2014; Lovejoy et al., 2014; Schwartz et al., 2014a; Li et al., 2015). The isomerization of uridine into ψ improves the base stacking in RNAs by the formation of additional hydrogen bonds, which influences RNA secondary structure and increases the stability of RNA duplexes (Arnez and Steitz, 1994; Davis, 1995). Pseudouridylation was shown to have a strong impact on different aspects of cellular processes, including translation efficiency, splicing, telomere maintenance, and the regulation of gene expression (Mochizuki et al., 2004; Carlile et al., 2014; Schwartz et al., 2014a). This base modification is catalyzed by pseudouridine synthases (Pus) that act on their substrates by two distinct mechanisms. One of those mechanisms is the guide RNA-dependent pseudouridylation, in which H/ACA box snoRNAs target RNAs for pseudouridylation *via* specific sequence interactions between the snoRNAs and the target RNA. A specific enzyme present in the snoRNP (sno-ribonucleoprotein) particule catalyzes the uridine modification (dyskerin in human, Cbf5 in yeast; Duan et al., 2009; Liang et al., 2009). Alternatively, RNA-independent pseudouridylation requires stand-alone pseudouridine synthases (Pus) that directly catalyze ψ formation at particular target RNA (Yu et al., 2011; Carlile et al., 2014; Rintala-Dempsey and Kothe, 2017). Each enzyme has a unique specificity for its target RNA and modifies uridine in a certain consensus sequence. Pus enzymes are present in all kingdoms of life, evolutionary conserved and are categorized into six families, based on their consensus sequences: TruA, TruB, TruD, RluA, and RsuA. The sixth family member, Pus10, is exclusive to eukaryotes and archaea (Koonin, 1996; Kaya and Ofengand, 2003; Fitzek et al., 2018).

Several pieces of evidence hint toward an implication of ψ in regulating neuronal functions. For instance, patients with mild-to-moderate severity of Alzheimer's disease show significantly elevated levels of urinal ψ (Lee et al., 2007) but it is currently unknown whether there is a link between this increase and the Alzheimer's disease etiology. Furthermore, it has been suggested that pseudouridylation can serve as a direct indicator of oxidative stress, which in turn has been linked to an increasing risk of neurodegeneration (Roth et al., 1999; Uttara et al., 2009). Accordingly, in cells exposed to acute oxidative stress by H_2_O_2_ treatment, Li and colleagues detected an elevation by ~40–50% in mRNA ψ levels, demonstrating that mRNA pseudouridylation acts as a direct response to cellular stress (Li et al., 2015).

A recent report demonstrated a direct implication of ψ in neuronal disorders from patients with myotonic dystrophy type 2 (DM2) (Delorimier et al., 2017). DM2 is a neuromuscular disease characterized by severe gray matter changes, including neuronal loss and global neuronal impairment (Minnerop et al., 2011; Meola and Cardani, 2015). DM2 patients have an increased binding of Muscleblind-like 1 protein (MBNL1) to CCUG repeats in an intron of the *CNBP* gene (Cho and Tapscott, 2007). Interestingly, it was recently reported that pseudouridylation within CCUG repeats reduces RNA flexibility and thus modestly inhibits MBNL1 binding (Delorimier et al., 2017). Similarly, ψ modification of a minimally structured model RNA resulted in an even more drastic reduction of MBNL1 binding to CCUG repeats. This study shows that ψ can reduce the disease-causing binding of MBNL1 at extended CCUG repeats and offers a basis for future research in treating neurodegenerative diseases.

### Pus1

Pus1 is a member of the TruA family that typically pseudouridylates tRNA but was also recently found to act on rRNA, snRNA, and mRNA (Schwartz et al., 2014a; Carlile et al., 2015). Mutations of *Pus1* in human lead to mitochondrial myopathy and sideroblastic anemia (Bykhovskaya et al., 2004; Fernandez-Vizarra et al., 2007; Bergmann et al., 2010). Recently, a mild cognitive impairment was also characterized in a long-surviving patient with two novel *Pus1* mutations (Cao et al., 2016). A different study demonstrated a Pus1-dependent pseudouridylation of the steroid RNA activator (SRA). Pseudouridylated SRA acts as a co-activator of the nuclear estrogen receptor α (ERα) (Zhao et al., 2004; Leygue, 2007). Given that ERα was shown to regulate neuronal survival (Gamerdinger et al., 2006; Foster, 2012), it is conceivable that one of the functions of Pus1 in brain activity is mediated *via* the control of the ER pathway.

### Pus3

Pus3 is another member of the TruA family, which has a strong sequence homology to Pus1 but acts on distinct target RNA. *In situ* hybridization showed accumulation of *Pus3* mRNA in the nervous system of mice embryos, suggesting a role of Pus3 in neural development (Diez-Roux et al., 2011). Accordingly, a truncated form of Pus3 accompanied by reduced levels of ψ U39 in tRNA was detected in patients with ID (Shaheen et al., 2016). Taken together, the discovery of impaired cognition caused by mutations of TruA enzymes emphasizes their importance in neuronal development and maintenance regulation.

### Dyskerin and RluA-1

The pseudouridine synthase dyskerin is essential for the H/ACA-box mediated pseudouridylation in human (Heiss et al., 1998; Lafontaine et al., 1998). Mutations of the dyskerin-encoding gene DKC1 causes X-linked recessive dyskeratosis congenita (DKC), a rare progressive congenital disorder that mostly affects highly regenerative tissues, such as the skin and bone marrow (Heiss et al., 1998; Mochizuki et al., 2004). Cells of the affected patients have decreased telomerase activity and thus reduced telomere length, which may be responsible for the disease (Mitchell et al., 1999). Interestingly, expression analysis of *Dyskerin 1* showed a high level in embryonic neural tissue, as well as in specific subsets of neurons in the cerebellum and olfactory bulb of adult brains (Heiss et al., 2000). While the function of dyskerin in hematopoiesis has been studied intensively, we are yet lacking a detailed understanding about its potential nervous system function in adult brains. In *Drosophila melanogaster*, RluA enzymes modify uridines in rRNA and tRNA. *In situ* hybridization in embryos revealed a specific RluA-1 mRNA localization to dendrites of a subset of peripheral neurons, which also raises the question about the molecular function of RluA-1 and its target RNA in the peripheral nervous system during embryonic development (Wang et al., 2011).

## N6-methyladenosine (m^6^A)

m^6^A is an abundant mRNA modification that regulates nearly all aspects of mRNA processing including splicing, export, translation, stability, and decay (Meyer and Jaffrey, 2017; Roignant and Soller, 2017; Roundtree et al., 2017). This modification is catalyzed by a stable protein complex composed of two methyltransferases, Methyltransferase like-3 (Mettl3) and Methyltransferase like-14 (Mettl14) (Sledz and Jinek, 2016; Wang et al., 2016a,b; Schöller et al., 2018). Additional proteins required for m^6^A deposition are Wilms‘ tumor 1-associating protein (Wtap) (Liu et al., 2014; Ping et al., 2014; Wang et al., 2014), Vir like m^6^A methyltransferase associated (Virma) (Schwartz et al., 2014b; Yue et al., 2018), Zinc finger CCCH domain-containing protein 13 (Zc3h13) (Guo et al., 2018; Knuckles et al., 2018; Wen et al., 2018), RNA binding protein 15 (Rbm15) and its paralog Rbm15B (Patil et al., 2016). Mettl3 has the catalytic activity and can accommodate the SAM substrate, while Mettl14 serves to stabilize the binding to RNA (Sledz and Jinek, 2016; Wang et al., 2016a,b; Schöller et al., 2018). In vertebrates, m^6^A modification is dynamically regulated and can be reversed by two demethylases belonging to the family of α-ketoglutarate dependent dioxygenases, Fat mass and obesity associated protein (FTO) and ALKBH5 (Jia et al., 2011; Zheng et al., 2013). Recent advances in techniques to map m^6^A modification in a transcriptome wide manner enabled identification of thousands of modified mRNAs and lncRNAs (Dominissini et al., 2012; Meyer et al., 2012). While m^6^A has been involved in many physiological processes, increasing evidence suggests an importance of m^6^A modification in brain development and in the function of the nervous system.

### m^6^A writer complex

m^6^A levels are particularly high in the nervous system, as shown in the developing mouse brain (Meyer et al., 2012), and in heads of adult flies (Lence et al., 2016). Furthermore, a recent study detected higher m^6^A content in the mouse cerebellum and in neurons compared to glia (Chang et al., 2017). Using *in situ* hybridization in zebrafish embryos, Ping et al. showed that Wtap is ubiquitously expressed at 36 h post-fertilization with enrichment in the brain region (Ping et al., 2014). Consistently, Wtap depletion using morpholino treatment resulted in severe developmental defects, including appearance of smaller brain ventricles and curved notochord at 24 h post-fertilization. Importance of m^6^A during neuronal development was further demonstrated by depletion of METTL3 in human embryonic stem cells (hESC), which strongly impaired neuronal differentiation (Batista et al., 2014), as well as the formation of mature neurons from embryoid bodies (Geula et al., 2015). Notably, m^6^A mRNA modification is essential for mouse survival as mice lacking Mettl3 die at E6.5 (Geula et al., 2015). However, two recent studies performed a conditional KO (cKO) of Mettl14 specifically in neurons and revealed an essential role of m^6^A in embryonic cortical neurogenesis (Yoon et al., 2017; Wang et al., 2018). Mettl14 cKO animals showed a decreased NSC proliferation and premature differentiation of NSCs (Wang et al., 2018), as well as delayed specification of different neuronal subtypes during brain development (Yoon et al., 2017). Yoon at al. further demonstrated that m^6^A modification is required for timely decay of transcripts involved in stem cell maintenance and cell cycle regulation in cortical neuronal progenitors. This allows accurate progression of the cell cycle and in turn induces the spatiotemporal formation of different neuronal subtypes. Interestingly, the authors also observed that many transcripts linked to mental disorders (autism, schizophrenia) are m^6^A modified in human, but not in mouse cultures of neuronal progenitor cells (NPC), raising the possibility that m^6^A regulates specifically these human diseases (Yoon et al., 2017). Consistent with this hypothesis, polymorphisms in *ZC3H13* have been associated with schizophrenia (Oldmeadow et al., 2014).

Beyond the role in neuronal development, m^6^A modification also plays a critical role in the process of axon regeneration in mature mouse neurons (Weng et al., 2018). Weng et al. showed that upon peripheral nerve injury m^6^A levels of many transcripts in dorsal root ganglion (DRG) were elevated, which led to increased translation during the time of axon regeneration, *via* the specific m^6^A reader protein Ythdf1. Mettl14 and Ythdf1 conditional KO mice displayed strong reduction of sensory axon regeneration, resulting from reduced protein synthesis, revealing the critical role of m^6^A modification in response to injury (Weng et al., 2018).

In *Drosophila*, loss of components of the methyltransferase complex results in severe locomotion defects due to altered neuronal functions (Haussmann et al., 2016; Lence et al., 2016; Kan et al., 2017). *Mettl3* mutants display alterations in walking speed and orientation, which can be rescued by ectopic expression of Mettl3 cDNA in neurons. Whether a particular subset of neurons is responsible for the observed alterations awaits further investigations. Interestingly, another member of the m^6^A methyltransferase complex, Nito (RBM15 in human), was recently shown to control axon outgrowth, branching and to regulate synaptic bouton formation *via* the activity of the CCAP/bursicon neurons (Gu et al., 2017), providing first insights toward addressing this question.

### m^6^A readers

As mentioned above, most characterized functions of m^6^A rely on the direct binding of the so-called m^6^A “reader” proteins to the modified site. The best-studied m^6^A readers are the YTH-domain containing proteins, which can specifically bind m^6^A *via* their YTH domain (Luo and Tong, 2014; Theler et al., 2014; Xu et al., 2014). RNA *in situ* hybridization of rat brain sections showed that one particular member of the YTH family, Ythdc1, is enriched in specific cells in the brain (Hartmann et al., 1999). Interestingly, in a yeast two-hybrid screen to identify Ythdc1-interacting proteins, libraries from P5 and E16 brains were screened and the rat homolog of Sam68 was found as the main interactor (Hartmann et al., 1999). Sam68 is known to regulate neuronal activity-dependent alternative splicing events (i.e., of neurexin-1) (Iijima et al., 2011). In line with this function, *in situ* hybridization assays showed that *Drosophila Ythdc1* localizes in the ventral neurectoderm and central nervous system of *Drosophila* embryos (Lence et al., 2016) and a reduced level of Ythdc1 was found to enhance SCA1-induced neurodegeneration (Fernandez-Funez et al., 2000).

Apart from the YTH-protein family of conventional m^6^A reader proteins, a number of other proteins that bind RNA in m^6^A-dependent fashion has been recently identified (Edupuganti et al., 2017). Among them, Fragile X mental retardation protein (FMRP, also known as POF; FMR1; POF1; FRAXA) was shown to preferentially bind an RNA probe containing m^6^A sites (Edupuganti et al., 2017). FMRP plays critical roles in synaptic plasticity and neuronal development. Its loss of function in human leads to the Fragile X syndrome, which is the most prevalent form of inherited ID and the foremost monogenic cause of autism (Bardoni et al., 2001; Lubs et al., 2012; Wang et al., 2012; Hagerman and Polussa, 2015). FMRP has a central role in neuronal development and synaptic plasticity through the regulation of alternative mRNA splicing, mRNA stability, mRNA dendritic transport and postsynaptic local protein synthesis of a subset of mRNAs (Antar et al., 2006; Didiot et al., 2008; Bechara et al., 2009; Ascano et al., 2012; Guo et al., 2015). Moreover, it represses mRNA translation during the transport of dendritic mRNAs to postsynaptic dendritic spines and activates mRNA translation of a subset of dendritic mRNAs at synapses (Bechara et al., 2009; Fähling et al., 2009). Consistent with a potential interplay between FMRP and m^6^A, a recent study found that m^6^A is present on many synaptic mRNAs that are known targets of FMRP protein (Chang et al., 2017). Future research will seek to further illuminate the potential role of FMRP within the m^6^A pathway. It is interesting to note that Nm also appears to contribute to FMRP-mediated translation regulation at synapses. FMRP can form a complex with the non-coding RNA, *brain cytoplasmic RNA* (*BC1*), to repress translation of a subset of FMRP target mRNAs (Zalfa et al., 2003). This interaction is modulated by the Nm status of *BC1* RNA. In both nucleus and cytoplasm in the cell body, Nm is present on *BC1*, but it is virtually absent at synapses (Lacoux et al., 2012). The authors suggested that changes in the 2′-O-methylation status of *BC1* RNA contribute to the fine-tuned regulation of gene expression at synapses and consequently to neuronal plasticity by influencing FMRP local translational control. This example supports a likely combinatorial role of RNA modifications in the regulation of similar targets and/or processes during brain function.

### m^6^A erasers

Two proteins in humans were reported to act as m^6^A erasers: (FTO) and AlkB homolog 5 (ALKBH5) (Jia et al., 2011; Zheng et al., 2013). Both belong to the family of Fe^2+^-α-ketoglutarate-dependent deoxygenases and catalyze the removal of the methyl group of m^6^A by oxidation. Interestingly, even though ALKBH5 is only moderately expressed in the brain, it has been associated with mental disorders. Du et al. found that certain polymorphisms within the *ALKBH5* gene correlate with the major depressive disorder (MDD), suggesting an involvement of ALKBH5 in conferring risk of MDD (Du et al., 2015).

In comparison, FTO is highly expressed in the human brain, especially in the hypothalamus and the pituitary gland and displays dynamic expression during postnatal neurodevelopment. Polymorphic alleles of *FTO* in human were identified to increase the risk for hyperactive disorder (Choudhry et al., 2013), for Alzheimer's disease (Keller et al., 2011; Reitz et al., 2012) and to affect the brain volume (Melka et al., 2013; Li et al., 2017a). Molecular and functional studies have shown that *FTO* knockout mice display altered behavior, including locomotion defects, but also influences learning and memory. For instance, fear conditioned mice showed a significant increase in m^6^A intensity on several neuronal targets, and knockdown of FTO further enhanced consolidation of cued fear memory (Widagdo et al., 2016). In line with this finding, *FTO* deficiency reduces the proliferation and neuronal differentiation of adult NSCs, which leads to impaired learning and memory (Li et al., 2017a). Electrophysiological tests in *FTO* knockout mice demonstrated an impaired dopamine type 2 and 3 receptor response, resulting in an abnormal response to cocaine (Hess et al., 2013). Strikingly, a recent study in mouse embryonic dorsal root ganglia found that FTO is enriched and specifically expressed in axons, influencing translation of axonal mRNAs (Yu et al., 2018). This demonstrates the dynamic role of m^6^A modification in regulating local translation. However, it is important to stress that FTO was also demonstrated to additionally demethylate m^6^Am, which is present next to the 7mG cap modification (Mauer et al., 2017). In comparison to m^6^A, m^6^Am also contains a methyl group on the ribose. As available antibodies recognize both modifications indistinguishably, it is currently difficult to assign their respective contribution in the context of brain activity.

## Conclusion

The association of aberrant RNA modifications with various neurological disorders highlights the importance of these chemical moieties for proper brain development and cognition. However, today the role of RNA modifications in these processes is not completely understood. One of the current challenges lies in identifying the class and identity of RNAs that are targeted by RNA modification enzymes and are causative of the neurological defects. Common targets for many of these enzymes are tRNAs and rRNAs, thus it is likely that in many cases their dysfunction plays an important role in the etiology of the disease. Yet, this does not explain why the phenotypes observed upon mutations of these enzymes are often restricted to the brain. Some of these enzymes or snoRNAs are predominantly expressed in the nervous system, which indicates their importance in this tissue and suggests the existence of differentially modified ribosomes (also recently called specialized ribosomes) that may carry distinct functions (Briggs and Dinman, 2017; Sloan et al., 2017). However, other snoRNA or enzymes exhibit wider tissue distribution, suggesting that brain specific phenotypes may reflect a higher sensitivity to altered translation in this tissue compared to others organs. This would be consistent with other disease-causing mutations in ribosomal proteins or tRNA synthetase genes that also manifest their effect specifically in the nervous system (Antonellis et al., 2003; Antonellis and Green, 2008; Yao and Fox, 2013; Brooks et al., 2014). What could be the reason(s) behind this increased sensitivity? It has been observed that tRNA level is in general higher in the brain compared to other tissues, suggestive of a bigger translational demand (Dittmar et al., 2006). This neuronal specificity may arise from the local translation that occurs at synapses upon environmental changes. In this context, RNA modifications represent an attractive system to regulate this acute need in a dynamic and flexible manner. However, other examples point toward a translation-independent mechanism. For instance, in the case of *FTSJ1* mutations and the associated NSXLID, neither the amount, nor the charging of the concerned tRNAs appear to be affected (Guy et al., 2012, 2015), seemingly ruling out a general translation defect. Perhaps in this case, the absence of modification can stimulate tRNA cleavage and generate tRFs that can also interfere with translation. This increase in tRFs would not necessarily be associated with a corresponding reduction of the uncleaved tRNA since tRNA levels are tightly regulated (Wilusz, 2015). Alternatively, the absence of modification on the tRNA may affect its interaction with the ribosome and thus influence translation efficiency or fidelity. Therefore, careful examination at different molecular levels is required to appreciate the effect of tRNA and rRNA modification enzyme mutations on translation and their consequence on the neurological phenotype.

**Figure 2:**
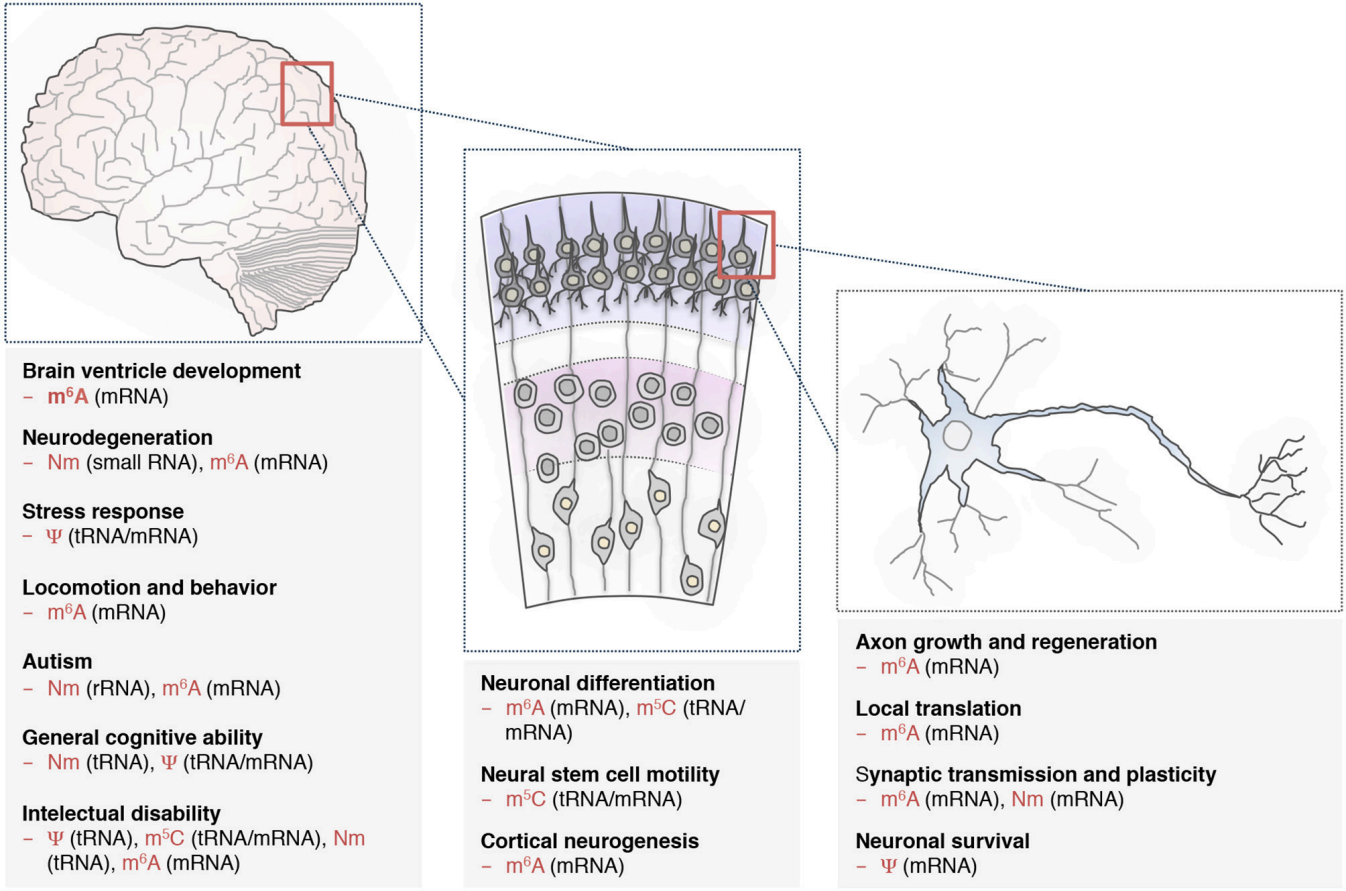
RNA modifications are implicated in various neuronal processes. Distinct RNA modifications of tRNAs, small RNAs and mRNAs are required for common biological processes during brain development **(left)**, neuronal differentiation **(middle)**, and proper functioning of individual neuron **(right)**, (see also Table 1). N6-methyladenosine (m^6^A), pseudouridine (Ψ), 5-methylcytosine (m^5^C), and 2′-O-methylation (Nm). The RNA classes in the brackets are the ones studied so far. Additional types with important functions may be modified as well.

That being said, the general picture is probably more complex. For instance, recent reports show that tRFs not only interfere with translation but can also affect transposon regulation and genome stability (Durdevic et al., 2013; Martinez et al., 2017; Schorn et al., 2017; Zhang et al., 2017). This activity could in principle also contribute to neurological disorders as growing evidence suggests associations between (re)expression of transposable elements and the occurrence of neuropathies (Perrat et al., 2013; Krug et al., 2017; Zahn, 2017; Jacob-Hirsch et al., 2018). In addition, beyond tRNA and tRF, the recent studies on m^6^A clearly demonstrate the involvement of mRNA modification in different aspects of neuronal development and regulation. The large diversity of RNA processing events in the brain, including the high rates of alternative splicing and recursive splicing (Duff et al., 2015; Sibley et al., 2015), the inclusion of microexons (Irimia et al., 2014) and the biogenesis of circular RNAs (cirRNAs) can all in principle be affected by RNA modifications. For instance, m^6^A modification was recently found on circRNAs, and enables their translation (Yang et al., 2017; Zhou et al., 2017). Given that misexpression of circRNAs has been associated with neurological disorders (Shao and Chen, 2016; van Rossum et al., 2016; Li et al., 2017b), some of m^6^A brain functions may rely on circRNA-mediated regulation. Thus, because neurons face distinct challenges with regards to localisation of RNAs to distal processes and to localized translation it is likely that in the brain, more than in any other tissues, a combinatorial effect of RNA modifications on different classes of RNAs represents a critical informational layer that dynamically fine-tunes gene regulation (Figure 2). Exciting discoveries are lying ahead for deciphering this intricate epitranscriptomics code.

## Author contributions

All authors listed have made a substantial, direct and intellectual contribution to the work, and approved it for publication.

### Conflict of interest statement

The authors declare that the research was conducted in the absence of any commercial or financial relationships that could be construed as a potential conflict of interest.
